# Newborn Screening Alone Cannot Prevent Most Cases of Severe Vitamin B12 Deficiency in the First Year of Life

**DOI:** 10.3390/nu17223583

**Published:** 2025-11-16

**Authors:** Christina Kaufman, Julian Margreitter, Marion Herle, Walter Bonfig, Corinne Däster, Bianka Heinrich, Daniela Karall, Hubert Kogler, Vassiliki Konstantopoulou, Alexander Laemmle, Reta Malär, Pascal Müller, Veronika Pöll, Martin Poms, Franziska Righini-Grunder, Rotraud K. Saurenmann, Susanna Sluka, Nicolas von der Weid, Maximilian Zeyda, Matthias R. Baumgartner, Martina Huemer

**Affiliations:** 1 Division of Metabolism and Children’s Research Center, University Children’s Hospital of Zurich, University of Zurich, 8008 Zurich, Switzerland; christina.kaufman@kispi.uzh.ch (C.K.); matthias.baumgartner@kispi.uzh.ch (M.R.B.); 2 Department of Child and Adolescent Health, Division of Pediatrics I—Inherited Metabolic Disorders, Medical University of Innsbruck, 6020 Innsbruck, Austria; julian.margreitter@i-med.ac.at (J.M.); daniela.karall@i-med.ac.at (D.K.); 3 Austrian Newborn Screening, Comprehensive Center for Pediatrics, Department of Pediatrics and Adolescent Medicine, Medical University of Vienna, 1090 Vienna, Austria; marion.herle@meduniwien.ac.at (M.H.); vassiliki.konstantopoulou@meduniwien.ac.at (V.K.); maximilian.zeyda@meduniwien.ac.at (M.Z.); 4 Department of Pediatrics, Klinikum Wels, 4600 Wels, Austria; walter.bonfig@klinikum-wegr.at; 5 Department of Pediatrics, Technical University Munich, 80333 Munich, Germany; 6 Neonatology, Kantonsspital Aarau, 5001 Aarau, Switzerland; corinne.daester@ksa.ch; 7 Department of Pediatrics, Stadtspital Zürich Triemli, 8063 Zurich, Switzerland; bianka.heinrich@stadtspital.ch; 8 Department of Paediatrics and Adolescent Medicine, St. Anna Children’s Hospital, Medical University of Vienna, 1090 Vienna, Austria; hubert.kogler@stanna.at (H.K.); veronika.poell@stanna.at (V.P.); 9 Division of Pediatric Endocrinology, Diabetology and Metabolism, Department of Pediatrics, Inselspital, Bern University Hospital, University of Bern, 3010 Bern, Switzerland; alexander.laemmle@insel.ch; 10 Department of Pediatrics, Cantonal Hospital Graubuenden, 7000 Chur, Switzerland; reta.malaer@ksgr.ch; 11 Pediatric Gastroenterology and Nutrition, Children’s Hospital of Eastern Switzerland, 9006 St. Gallen, Switzerland; pascal.mueller@kispisg.ch; 12 Newborn Screening Switzerland, University Children’s Hospital Zurich, University of Zurich, 8008 Zurich, Switzerland; martin.poms@kispi.uzh.ch (M.P.); susanna.sluka@kispi.uzh.ch (S.S.); 13 Division of Pediatric Gastroenterology, Hepatology, and Nutrition, Department of Pediatrics, Children’s Hospital of Central Switzerland, 6000 Lucerne, Switzerland; franziska.righini@luks.ch; 14 Division of Pediatric Gastroenterology, Hepatology, and Nutrition, Department of Pediatrics, University Children’s Hospital Zurich, 8008 Zurich, Switzerland; 15 Center of Pediatrics, Cantonal Hospital Winterthur, 8401 Winterthur, Switzerland; traudel.saurenmann@ksw.ch; 16 University Children’s Hospital Basel (UKBB), University of Basel, 4031 Basel, Switzerland; nicolas.vonderweid@ukbb.ch; 17 Department of Pediatrics, LKH Bregenz, 6900 Bregenz, Austria; 18 Vorarlberg University of Applied Sciences, Competence Area Healthcare and Nursing, 6850 Dornbirn, Austria

**Keywords:** newborn screening program, cobalamin, propionylcarnitine, homocysteine, methionine

## Abstract

Background/Objectives: Vitamin B12 (B12) is essential for the provision of methyl groups for numerous essential pathways. Infant B12 deficiency (B12D) can lead to severe, even irreversible neurological abnormalities. Maternal B12 status in pregnancy and during the breastfeeding period correlates significantly with the child’s B12 status. B12D is a target disease in some newborn screening (NBS) programs. This study investigates whether infants that were clinically symptomatic and diagnosed with B12D in their first year of life could be retrospectively detected by the Austrian NBS algorithm. Methods: Data from infants with clinically diagnosed B12D in their first year of life between 2012 and 2022 were retrospectively collected in Austria (B12-related NBS implemented in 2018) and Switzerland (B12-related NBS not implemented). NBS data were retrospectively analysed, and clinical information was collected by a survey. Correlations between clinical symptoms, NBS data, biochemical parameters at diagnosis, maternal medical history and B12 status were analysed. Results: Four/forty-eight cases were retrospectively detected by the first-tier NBS parameters. From two children material for second-tier testing was available and B12D was confirmed by elevated total homocysteine (tHcy), resulting in a detection rate between 4.3 and 9.3%. The numbers of neurological and haematological symptoms correlated with low B12 and elevated levels of tHcy and methylmalonic acid. Although the detection rate of symptomatic B12D by NBS was low, fewer infants with symptomatic B12D were observed in the period after implementation of B12-related NBS (Austria). A history of B12D-relevant maternal disease such as pernicious anaemia was reported in 12 cases. Conclusions: B12D causes severe clinical symptoms in infants. NBS has a very limited retrospective detection rate of infants with severe B12D but seems to correlate with a reduction in cases due to not yet precisely quantified mechanisms. The workup triggered by NBS recalls is costly and often challenging for families. Maternal B12D increases the risk of infant B12D but also of other pregnancy-related health risks. To increase the efficacy of the prevention of infant B12D, to promote a healthy pregnancy and breastfeeding period, and to reduce the frequency of NBS recalls, pregnant women should be screened for B12D to be counselled and treated.

## 1. Introduction

Vitamin B12, or cobalamin (Cbl), is an essential micronutrient that plays an important role in cellular metabolism acting as cofactor for two metabolic enzymes. After gastrointestinal absorption and complex intracellular processing steps, Cbl as methylcobalamin is a cofactor of the cytosolic methionine synthase (MTR), and Cbl as adenosylcobalamin is a cofactor for the mitochondrial methylmalonyl-CoA mutase (MMUT) [1]. MTR remethylates methionine from homocysteine (Hcy) and provides methyl groups for numerous metabolic reactions such as DNA, RNA, phospholipid and neurotransmitter synthesis. This reaction depends on the provision of methyl groups via the folate cycle [2].

Homocysteine remethylation to methionine is additionally and independently from the B12 pathway performed by the enzyme betaine-homocysteine-S-methyltransferase via betaine as the methyl donor and with choline as a substrate [3]. Choline is frequently a critical nutrient in pregnant women [4].

**Figure 1:**
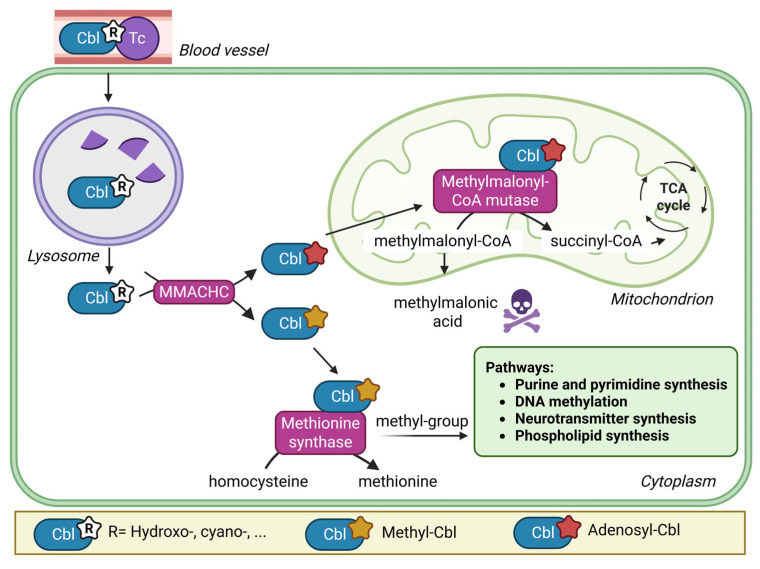
Simplified pathway of intracellular cobalamin processing. Legend: In blood Cbl is bound to the transport protein transcobalamin (Tc). The Cbl/Tc complex (holoTC) is taken up by the cells via receptor-mediated endocytosis. In the lysosome Tc is degraded and Cbl is liberated. In the cytoplasm, functional groups are removed, Cbl is reduced and the bioactive forms methyl-Cbl and adenosyl-Cbl are synthesised. Created in BioRender. Forny, P. (2025) https://BioRender.com/6443h9d (accessed on 20 August 2025).

MMUT is needed for the intramitochondrial conversion of methylmalonic acid (MMA) into succinyl-coA (Figure 1) [2].

The gastrointestinal absorption of Cbl from food is impaired with the chronic use of proton pump inhibitors [5] and in acquired disorders such as pernicious anaemia, chronic inflammatory bowel or coeliac disease, post bariatric surgery [6] or in genetic conditions such as congenital intrinsic factor deficiency [7]. The intracellular processing of Cbl is disrupted in several rare inherited metabolic disorders (e.g., the cblC-*MMACHC* defect) [8].

Food of animal origin is the primary source of vitamin B12 in humans [9], and insufficient intake is the most prevalent origin of B12 deficiency (B12D) in healthy individuals [10,11,12,13].

The focus of this study is severe, symptomatic B12D in the first year of life caused by insufficient maternal and nutritional Cbl supply. In the first weeks and months of life, infant and maternal B12-related metabolites correlate strongly. Children born to mothers adhering to a vegan or vegetarian diet without supplementing B12 or with unknown Cbl malabsorption or trafficking disorders have a significantly increased risk of B12D due to low stores at birth and impaired B12 supply during breastfeeding [14,15].

Holo-transcobalamin (holoTC) is the active form of Cbl that enters the cells. As explained by its intracellular targets, impaired Cbl bioavailability causes elevated MMA and total Hcy (tHcy) blood concentrations. B12 is almost always assessed, but MMA, tHcy, and holoTC are not as widely used [16]. Methionine is the weakest parameter, as it may be low or remain normal [1].

The optimal indicators and their cutoffs for the diagnosis of B12D have so far not been uniformly defined, especially not for infants. The interpretation of reference intervals or cutoffs must not only be age-related but must consider differences in measurement methods, population sizes, genetic and nutritional backgrounds and calculation models. In clinical practice, B12 < 148 pmol/L is often used as a cutoff for B12D, and 149–221 pmol/L for a low B12 status. However, these reference values have been established in adults [17] and are not without controversy [18]. Reference ranges and cutoffs have so far only been investigated in small and age-diverse paediatric populations. In a Norwegian population the 2.5 percentile of total B12 was 295 (pmol/L in six- to eight-year-old and 249 pmol/L in 9- to 12-year-old children) [19]. The extent to which such references can be transferred to other populations and age groups is unclear. The assessment of plasma B12 does not differentiate between haptocorrin-bound cobalamin and holoTC [17]. For adults, a normal range for holo-TC has been established at 20–125 pmol/L [20]. In the Norwegian sample 56 and 37 pmol/L represented the 2.5th percentile [19]; in a small cohort of younger children, the 2.5th percentile varied with age between about 30 and 45 pmol/L [21].

Serum/plasma MMA concentrations have been considered elevated from >260, >300 or >350 nmol/L [17]. The 97.5th percentile value in a Danish sample of 393 children was 360 nmol/L [18]. Plasma tHcy concentrations of >6.5 [22], >10 [23] and >13 [17] have been suggested as upper cutoffs. Given this variability and lack of clarity as to which cutoffs are best suited to the population under investigation, the clinical diagnosis of B12 deficiency was set as the criterion for inclusion in this study.

In accordance with the significance of the remethylation pathway as the main provider of methyl groups for numerous essential pathways, infant B12D can lead to severe neurological abnormalities such as irritability, feeding difficulties, failure to thrive, developmental delay or loss of achievements [10,24]. Infant B12D may even result in irreversible nervous system damage and brain atrophy. Beyond the neurological symptoms (macrocytic), anaemia is frequent [11,25].

**Figure 2:**
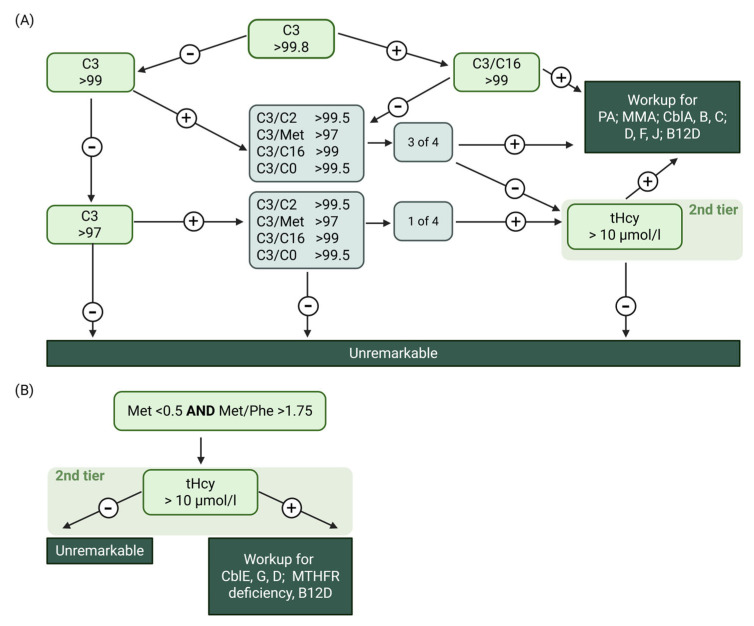
The Austrian first- and second-tier NBS algorithm to identify B12D. (**A**) Propionylcarnitine (C3) path, (**B**) Methionine path. Created in BioRender. Forny, P. (2025) https://BioRender.com/6443h9d (accessed on 20 August 2025).

Since June 2018 the Austrian neonatal screening programme (NBS) targets inborn errors of the Cbl pathway (Figure 2). To identify these, methionine, propionylcarnitine (C3) and ratios such as C3/methionine and C3/acetylcarnitine are used as first-tier parameters [12,26]. THcy is the second-tier parameter [27]. These parameters can also be out of range when B12 availability is low.

It is so far unclear whether newborns with clinically diagnosed B12D in infancy are a subgroup of the neonates detected by NBS with low B12 concentrations. Consequently, it is unclear whether early treatment of NBS-identified neonates with low B12 status prevents severe clinical B12D. Thus, it is still under discussion whether detecting and treating children with suboptimal neonatal B12 status is a valuable additional NBS benefit preventing serious harm [28,29].

To address this topic, this study focuses on the question of whether infants that were clinically symptomatic in their first year of life and diagnosed with B12D would have been detected by the Austrian NBS algorithm to allow for preventive treatment. To this purpose, data from infants with clinically diagnosed B12D in their first year of life between 2012 and 2022 were retrospectively collected in Switzerland and Austria and their NBS dried blood spots (DBS)/data were analysed according to the algorithm of the Austrian NBS.

## 2. Methods

The study was conducted in compliance with ethical standards and received approval from the Ethics Committee of the Medical University of Innsbruck (Austria; EK Nr: 1149/2022) and the cantonal Ethics Committee Zurich (Switzerland; BASEC-Nr: 2022-01725). Due to the retrospective, pseudonymized character of the study, no individual consent was obtained. If in the Swiss centres general consent forms had been in use at the time of patient admission, they had to have been signed, which may result in a selection bias. Patients diagnosed with B12D before implementation of the general consent in the reporting institution were included based on the parents’ consent to NBS, which includes permission to perform research to improve and further develop the Swiss NBS. Data were pseudonymized to protect patient confidentiality. The study group only had access to pseudonymized data provided by the reporting centres and the NBS programme.

## 3. Patient Cohort

Data from infants clinically diagnosed with B12D in the first 12 months of life and confirmed by biochemical testing according to local standards between January 2012 and December 2022 were included in the study. Exclusion criteria were absence of clinical symptoms (e.g., diagnosis by family screening), unverified B12D or alternative diagnoses such as inborn errors of Cbl metabolism.

In Switzerland, certified paediatric training centres listed on “Schweizerisches Institut für ärztliche Weiter- und Fortbildung” (https://www.siwf.ch/; accessed on 1 November 2022) were contacted. In Austria, paediatric clinics and departments listed on the website of the Austrian Society of Paediatrics (https://www.paediatrie.at/; accessed on 1 June 2022) were contacted. Private practices or other institutions were not contacted. A total of 20 of 86 contacted paediatric units responded to our enquiry. A total 5 of 57 paediatric departments and clinics in Austria and 8 of 29 in Switzerland agreed to contribute clinical and biochemical data from 21 and 31 patients, respectively, with B12D diagnosed during the past 10 years. Four patients did not fulfil the inclusion criteria and were excluded, resulting in a study sample of 48 cases. Using a pseudonymized case questionnaire information on age, sex, anthropometric data at birth and at diagnosis, symptoms at diagnosis and outcome was collected. Percentiles were determined according to Fenton et al. [30] at birth and according to Kromeyer-Hauschild et al. [31] at diagnosis. If the gestational age for term-born children was unknown, it was set at 40 weeks’ gestation for percentile calculation.

Values of B12, holoTC, MMA, tHcy and methionine in blood and MMA in urine were obtained from tests performed in the laboratories of the reporting centres and were collected in their local units (local reference ranges were not collected). The units were converted by the study team for data harmonisation for further analysis (see Table 2). Additionally, type of diet and B12 supplementation at diagnosis were collected for both mother and child. Relevant medical history data of the mother (previous illnesses, bariatric surgery) were also obtained. The centres contacted their respective NBS centre directly to request the B12-related NBS raw data (Austria) or the re-evaluation of available raw data according to the current Austrian NBS algorithm (Switzerland). Notably, the use of percentile cut offs allows the utilisation of the algorithm in different labs independent of absolute values, which may differ from lab to lab due to technical reasons. Percentile distributions for the Swiss cohort were derived separately for each calendar year from all initial newborn screening card values to minimise inter-year variation and long-term drift. Analogous to the Austrian algorithm, screenings that would have triggered a second-tier tHcy measurement were considered positive if predefined percentile thresholds were exceeded. For the acylcarnitine-based criterion, results were classified as positive when C3 exceeded the 99th percentile, or when C3 was above the 95th percentile in combination with at least one of the following ratios above their respective thresholds: C3/Met (>99.5th percentile), C3/C16 (>99.5th percentile), C3/C2 (>99.8th percentile) or C3/C0 (>99.5th percentile). For the methionine-based criterion, screens were considered positive when methionine was below the 0.2nd percentile or when the methionine/phenylalanine ratio was below the 0.2nd percentile.

Pseudonymized Austrian and Swiss data sets were collected and managed using REDCap electronic data capture tools hosted at University Children’s Hospital of Zurich [32,33].

## 4. Statistical Analysis

First, descriptive analysis (mean, median, SD, IQR, range) of the study parameters (NBS data, age at first symptoms, anthropometric data at birth and at diagnosis; age, symptoms, nutrition and biochemical parameters of the child at diagnosis; treatment of the child, maternal diet at diagnosis, maternal health problems) was performed. Data were checked for content plausibility (CK, MH); outliers were identified during the descriptive analyses.

One-sided correlations were calculated between all biochemical parameters at diagnosis. Two-sided correlation analyses were performed between (a) NBS numerical data and biochemical parameters at diagnosis, (b) age at first symptoms and NBS numerical data, (c) biochemical parameters at diagnosis and total number of symptoms at diagnosis, (d) biochemical parameters at diagnosis and number of symptoms in each symptom category (haematological, failure to thrive, acute/chronic neurologic, movement, cognition) and (e) total number of symptoms and time between first symptoms and diagnosis.

Two-sided Median Test was used to compare the time between first symptoms and diagnosis between groups of children formed based on specific symptoms.

To compare the anthropometric data of the children between birth and time of diagnoses, z-scores for weight, length and head circumference were calculated. Paired T-Test (two-sided) was applied to compare these z-scores between the two time points.

Normality of the data was checked via Kolmogorov–Smirnov test, and linearity was explored via scatterplots. If the data were not normally distributed, the assumption of linearity was not tenable (could not be confirmed) or the variables were ordinal scaled, non-parametric methods (Spearman rank correlation, median test) were used. Missing cases were excluded pairwise. For the Spearman correlations, 95% CIs were computed using the Bonett and Wright method.

To investigate whether the number of children clinically diagnosed with B12D in their first year of life has decreased in Austria since the introduction of NBS for B12D, the observation period was divided into before (1 January 2011–31 May 2018) and after the introduction of NBS for B12D (1 June 2018–31 August 2023). The actual numbers of symptomatic children observed in the two periods was compared with the expected numbers (assuming an unchanged frequency of B12D before and after implementation of NBS for B12D) using one-tailed Chi-square test. For the Swiss cohort this calculation was performed with a two-sided *p*-value, as a decrease in the number of cases was not expected in this group. The Chi-square test was also used to explore whether the number of cases of symptomatic children in Austria and Switzerland had developed differently between the two study periods.

*p*-values ≤ 0.05 were considered statistically significant. *p*-values for correlations between biochemical parameters were adjusted using the Bonferroni–Holm method. Since the other analyses were of explorative character, correction for multiple testing was not applied, which might enhance the potential for type I errors. All calculations were performed with SPSS (IBM SPSS Statistics 29.0.2.0). Graphs were designed using Graph Pad Prism (Version 10.1.2 for Windows, GraphPad Software, Boston, MA, USA, www.graphpad.com; accessed on 15 June 2025). Figures were created with BioRender, (2025, Toronto, ON, Canada), www.biorender.com, accessed on 20 August 2025. 

## 5. Results

Forty-eight cases (42 born at term, 3 preterm, 3 missing data; 23 males) were included. At the time of B12D diagnosis, one infant was supplemented with oral 2 mg/d methylcobalamin, and three received iron preparations. One infant was treated with melatonin.

**Figure 3:**
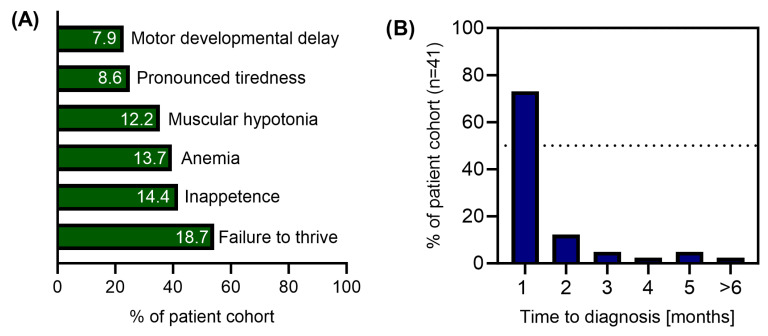
Major clinical symptoms in infants with vitamin B12 deficiency at presentation (**A**) and time between first symptoms and diagnosis (**B**).

Mean age at first symptoms of B12D was 5.2 months (median 6, range 0–12 months, n = 45) and age at diagnosis was 5.7 months (median 6, range 0–17 months, n = 43). The diagnosis of B12D was established in 85% of cases within two months from symptom onset. The distribution of clinical symptoms at presentation and the time to diagnosis are shown in Figure 3. Information on auxological parameters, infant and maternal diet and history data on maternal health problems are summarised in Table 1. Four children had further medical conditions that were not considered to cause B12D by the clinicians.

Numerical NBS parameters were available for 17 Austrian children and percentiles of NBS parameters for 26 Swiss children (n = 43). Age at first symptoms and NBS parameters did not correlate. Three Austrian children (born before B12D-NBS implementation) and one Swiss child would have been detected by the current Austrian NBS first-tier algorithm resulting in a first-tier diagnostic yield of 9.3% (n = 4/43 cases). Three cases were detected via the C3 path, one by low Met and Met/Phe. Material for retrospective second-tier testing was available for 2/4 first-tier positive cases and, in both, tHcy was above the cutoff.

Case 1: This female term-born child was detected by elevated C3 and the respective ratios and by positive second-tier testing (tHcy 10.8 µmol/L). She had a birthweight of 2840 g (−1.2 z), length of 49 cm (−0.95 z) and a head circumference of 33 cm (−1.23 z). At the age of 6 months, the infant presented with inappetence, repetitive vomiting and failure to thrive with percentile crossing (body weight: −1.87 z; head circumference: −1.62 z). Additionally, muscular hypotonia, lack of movement and a resulting developmental delay were noted. The diagnosis was confirmed with low B12 at 50 ng/L and elevated MMA in urine at 253 mmol/mol creatinine. At the time of diagnosis, the infant was exclusively breastfed and refused solid foods. The cause of the infant’s B12D was traced to a maternal deficiency caused by autoimmune gastritis.

Case 2: This male term-born was detected by elevated C3 and the respective ratios (material for second-tier testing not available). He had a birthweight of 2560g (−1.62 z), length of 49 cm (−1.06 z) and a head circumference of 34 cm (−0.9 z). At the age of 6 months, he presented with muscular hypotonia and anaemia. Auxological parameters were stable: weight: −1.64 z, length −0.96 z, head circumference 0.26. Vitamin B12 levels were very low (<22 ng/L) and urinary MMA was elevated (1220 mmol/mL creatinine). At the time of diagnosis, the boy was breastfed and received solid food. Maternal B12 was low with 178 ng/L.

Case 3: This term-born female was detected by elevated C3 and the respective ratios (material for second-tier testing not available). Her birthweight was 2635g (−1.45 z), and her weight at diagnosis was 4600g (−1.13 z). At the age of three months, she presented with vomiting, anaemia and fatigue. B12D was confirmed by low vitamin B12 levels of 50 ng/L. The girl was exclusively breastfed. The infant’s B12D was attributed to maternal pernicious anaemia.

Case 4: This male term-born with a birthweight of 3500g (0.42 z) and head circumference of 36cm (1.65 z) was detected by decreased Met and Met/Phe ratio. Material for second-tier testing was available and tHcy (15.4 µmol/L) was elevated. He developed muscular hypotonia, reduced appetite, failure to thrive (weight −1.49 z) and anaemia in the first month of life. Diagnosis was confirmed by low levels of vitamin B12 in blood (117 ng/L) and elevated MMA in urine (600 mmol/mol creatinine). The child received formula milk and breastmilk. His mother was diagnosed with autoimmune gastritis, and her B12 (61 ng/L) and holo-TC (6.1 pmol/L) concentrations were low.

The biochemical parameters at diagnosis are shown in Table 2. Two-tailed correlation analyses of NBS numerical data and parameters at diagnosis in the Austrian cohort (n = 17) showed significant correlations between higher C3, C3/Met, C3/C2 and C3/C0 in NBS and lower vitamin B12 in blood at diagnosis (Rho(15) = −0.53, *p* = 0.03, 95% CI [−0.82, −0.03]; Rho(15) = −0.57, *p* = 0.02, 95% CI [−0.84, −0.08]; Rho(15) = −0.62, *p* = 0.01, 95% CI [−0.86, −0.15]; Rho(15) = −0.55, *p* = 0.02, 95% CI [−0.83, −0.05]).

**Table 3:** Correlations of biochemical parameters at diagnosis and clinical symptoms (two-tailed p).

	Biochemical Parameters at Diagnosis				
	tHcy	MMA in Blood	MMA in Urine	Vit B12	holoTC
Total number of symptoms	ρ(23) = 0.71 (*p* = < 0.001) 95% CI [0.40, 0.88]	ρ(19) = 0.57 (*p* = 0.01) 95% CI [0.15, 0.82]	ρ(34) = 0.39 (*p* = 0.02) 95% CI [0.06, 0.64]	ρ(40) = −0.33 (*p* = 0.03) 95% CI [−0.58, −0.02]	
Acute neurological symptoms	ρ(23) = 0.54 (*p* = 0.01) 95% CI [0.15, 0.78]				
Chronic neurological symptoms	ρ(23) = 0.43 (*p* = 0.03) 95% CI (0.02, 0.72]				
Impaired motor performance	ρ(23) = 0.67 (*p* < 0.001) 95% CI [0.33, 0.85]	ρ(19) = 0.59 (*p* = 0.01) 95% CI [0.18, 0.83]	ρ(34) = 0.38 (*p* = 0.02) 95% CI [0.04, 0.63]	ρ(40) = −0.36 (*p* = 0.02) 95% CI [−0.60, −0.05]	
Cognitive impairment					ρ(12) = 0.61 (*p* = 0.02) 95% CI [0.06, 0.87]
Haematological symptoms	ρ(23) = 0.83 (*p* < 0.001) 95% CI [0.61, 0.93]		ρ(34) = 0.67 (*p* < 0.001) 95% CI [0.41, 0.83]	ρ(40) = −0.55 (*p* < 0.001) 95% CI [−0.74, −0.28]	

Higher MMA in urine correlated significantly with higher C3 (Rho(10) = 0.9, *p* < 0.001, 95% CI [0.59, 0.98]; C3/C2 Rho(10) = 0.71, *p* = 0.01, 95% CI [0.15, 0.92]; and C3/C0 Rho(10) = 0.75, *p* = 0.01, 95% CI [0.23, 0.94]). Higher C3/C2, C3/C0 at birth correlated significantly with higher tHcy at diagnosis (Rho(8) = 0.78, *p* = 0.01, 95% CI [0.20, 0.96]; Rho(8) = 0.65, *p* = 0.04, 95% CI [−0.05, 0.92]. Correlations between the NBS parameters and MMA in blood and holoTC were not evaluated due to the small numbers of cases (n = 4; n = 3).

One-tailed correlation between the parameters measured at diagnosis was calculated for the complete cohort of Austrian and Swiss children. As expected, low vitamin B12 correlated significantly with high tHcy (Rho(21) = −0.54, p_adj_. = 0.01, 95% CI [−1.00, −0.20]), MMA in blood (Rho(15) = −0.51, p_adj_.= 0.04, 95% CI [−1.00; −0.09]) and MMA in urine (Rho(28) = −0.68, p_adj_. = 0.01, 95% CI [−1.00, −0.44]). While blood and urinary MMA correlated significantly (Rho(14) = 0.69, p_adj_. = 0.01, 95% CI [0.33, 1.00]), tHcy correlated only with urinary MMA (Rho(20) = 0.46, p_adj_. = 0.04, 95% CI [0.10, 1.00]). HoloTC did not correlate with any of the other parameters.

The total number of symptoms did not correlate with the time between first symptoms and diagnosis. The time between first symptoms and diagnosis did not differ when groups of children with versus without a specific symptom (e.g., anaemia or muscular hypotonia; calculated for all symptoms) were compared. Weight z-scores were significantly lower at diagnosis than at birth (t(41) = 2.93, *p* = 0.01). This corresponds with the reported high frequency of failure to thrive and inappetence at diagnosis.

At diagnosis, 28 infants were fully and 14 partially breastfed. The suspected aetiology of infant B12 deficiency was reported for 21/48 cases. Maternal autoimmune/pernicious anaemia (n = 3), vegan nutrition with insufficient maternal B12 supply (n = 3) and unspecified maternal B12D (n = 15) were considered causal by the clinicians.

**Figure 4:**
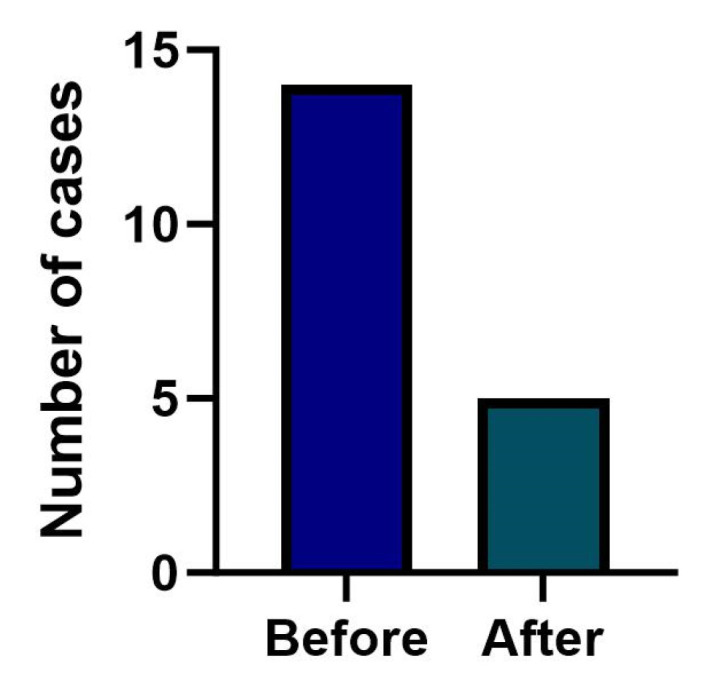
Numbers of clinically diagnosed infants with B12D before and after the implementation of B12-related NBS in Austria in June 2018.

The Austrian NBS algorithm for B12-related inborn errors of metabolism was implemented in June 2018. Birth rates in Austria and Switzerland were not significantly different between the periods from January 2011 to June 2018 and from July 2018 to August 2023. Assuming that the NBS algorithm does not prevent infants from developing clinically symptomatic B12D, that factors influencing B12 status do not differ significantly between the two countries and that the incidence of B12D remains stable, numbers of cases should be equally distributed before and after NBS implementation. However, the numbers of cases with symptomatic B12D before (1 January 2011–31 May 2018) and after (1 June 2018–31 August 2023) implementation of B12D-NBS in Austria (Figure 4) developed significantly different between the countries (Chi^2^ (1, *N* = 46) =10.01, two-sided *p* < 0.001). In the Austrian cohort the expected counts (11 before, 8 after implementation) and the observed counts (14 before, 5 after implementation; 95% CI [7.65–23.49]; 95% CI [1.62–11.67]) were not significantly different (Chi^2^ (1, *N* = 19= 1.79, one-sided *p* = 0.09, d = 0.65). In Switzerland the expected (16 before, 11 after June 2018) and observed counts (6 before, 21 after June 2018) were significantly different (Chi^2^ (1, *N* = 27) = 14.69, two-sided *p* < 0.001), indicating more observed cases in recent years.

Information on the treatment of the children with B12D was available for 45 cases. Forty children were treated with Cbl, most of them parenterally (n = 14 cyanocobalamin, n = 26 hydroxocobalamin). Oral cyanocobalamin was administered in two cases, and oral and intranasal methylcobalamin were used in one case each. Doses, application frequency and duration of treatment varied widely from a single dose to 2–7 doses of Cbl in the first week. Cbl treatment was repeated weekly or monthly in some children, and in single cases given for up to one year. In one case, only a switch from breast milk to formula was initiated. Treatment of the mothers was reported for eight cases (three parenterally, one oral, four missing information), no treatment for two cases and for the remaining cases, treatment status was unknown (n = 27) or data were missing (n = 11). Thirty-eight children recovered fully after treatment. Persistent developmental impairment was observed in four, persistent failure to thrive in one, inadequate length gain in one and muscular hypotonia in one patient(s) were noted (n = 3 missing data). No dose–effect or route of application–effect ratios were observed.

## 6. Discussion

In an earlier publication we reported on the positive predictive value of the Austrian NBS Algorithm for the detection of suboptimal neonatal B12 status in the Austrian population. Of 203.440 screened neonates, 7127 (3.5%) had a positive first-tier test, and in 0.079% (161 cases) the second-tier test tHcy was also above norms. Of these, 34% (55 cases) were diagnosed as B12D if they fulfilled at least one of the following criteria: serum B12 < 150 pmol/L or holoTC < 25 pmol/L or serum MMA > 600 nmol/L [16].

The current study was conducted to explore whether infants that were clinically symptomatic in their first year of life and diagnosed with B12D would have been detected by the Austrian NBS algorithm to allow for preventive treatment. The related question of whether the detection of newborns using this algorithm makes a significant contribution to the advantages of NBS in relation to resource consumption is also discussed.

In a similar study, DBS from 70 Norwegian children with B12D were retrospectively analysed according to the Austrian and the Heidelberg (Germany) algorithms [29]. Our study was conducted because the comparability of the Norwegian and the here-reported cohort must be considered limited, not only due to different genetic backgrounds, but also since in Norway, nitrous oxide (N2O) is frequently used for pain relief in mothers during labour. N2O is not widely used for pain relief in mothers during labour in Austria, but is used by some centres in Switzerland. However, exact information on its use is not available.

Through oxidation of the cobalt ion contained in cobalamin from its functional +1 oxidation state to a non-functional +3 state by N2O, the intracellular functions of methyl- and adenosylmethionine come to a standstill [34], causing an increase in MMA but—probably more importantly—an arrest of the Hcy-methionine cycle, the main donor of methyl groups that are crucial for the synthesis of myelin or the post-translational methylation of the DNA [35]. Thus, maternal N2O use during labour affects B12 bioavailability and functionality in both mother and child [36] and might result in earlier (infant age of 6 months in our cohort versus 10.9 weeks in Norway at workup for B12D) or more pronounced B12D. With the Austrian first-tier test set, four (5.7%) of the seventy Norwegian B12D cases could retrospectively be identified, one by the C3 and three by the Met algorithm. Two of these four cases were also second-tier test positive (tHcy > 6.3 µmol/L at the time). This diagnostic yield of 2.9% in this Norwegian population resembles the performance of the Heidelberg algorithm (4.3%) that had a higher rate of positive first-tier tests (18.6%), requiring tHcy assessment [29]. In this study, three cases were identified by the C3 and one by the Met algorithm. The different distribution of cases detected by the C3 and the Met algorithm between this and the Norwegian study may reflect the effect of N2O, which primarily inhibits methionine synthesis [34]. THcy was above norm in both cases, in which second-tier testing could retrospectively be conducted. This results in a retrospectively confirmed first- and second-tier detection rate of 4.3%. Since the retrospective detection rate of the first-tier test was 9.3% (4 out of 42 cases, with NBS data or material missing in 6 cases), this value corresponds to the maximum possible first and second-tier detection rate for this population, which is like the Norwegian study [29].

We studied the differences between the number of expected and the observed cases before and after implementation of B12D-targeting NBS and found a rise in case numbers in Switzerland, where NBS for B12D is not performed. Although this pattern is consistent with the possibility that B12D-targeting NBS may reduce the reporting of symptomatic B12D, interpretation must remain cautious. Limitations to this conclusion include probable ascertainment bias, different states of B12-related awareness over time and between countries, changing referral patterns, demographic shifts or missing information on the dietary habits and their changes in both countries, as well as small sample sizes. While the observation that clinically diagnosed B12D in the first year of life was reported four times more frequently in German populations without compared to populations with B12-related NBS [37] supports this hypothesis, it does not establish causality.

The balance between resource use and effect of a preventive measure becomes increasingly important in times of dwindling resources and rising costs in healthcare systems, and it must be carefully considered whether B12D should be included as a target disease in NBS programmes in future. Our data and the retrospective analysis of 70 Norwegian cases suggest that NBS has some, albeit very limited, effectiveness in identifying children who developed severe, clinically diagnosed B12D in their first year of life [29]. It was confirmed in this cohort that B12D is a treatable condition, although in the absence of standardised treatment protocols a substantial variability regarding dose, route and duration of B12 treatment was observed.

The many newborns also identified by the algorithm and not affected by inborn errors of the pathways seem to have a suboptimal B12 status with a not yet precisely quantified risk for health impairment.

The costs for an individual case workup and treatment triggered by NBS results indicative of B12D include state-of-the-art biochemical testing, successful treatment with hydroxocobalamin (and folate) and follow-up blood testing, which need to be considered and may vary between different countries or health systems. In an Italian cohort costs per case were calculated to be € 3578 [38]. As it is advised to perform reverse cascade testing of the mother following a NBS recall indicating the possibility of an underlying maternal disease or increased health risk [39], even higher costs per mother–child dyad must be assumed. Not only must the cost–benefit analysis be considered, but the acceptance of NBS and the protection of families from avoidable stress are also highly valued. A comprehensive psychological study used standardised methods to examine the emotional reactions and assessments of parents regarding their child’s health after they had been called back to one of the Italian metabolic centres to be informed of a positive NBS result. In this study, 80% of 169 fathers and 171 mothers reported a clinical level of distress and anxiety, some even experienced post-traumatic symptoms and a majority wished for psychosocial support without differences between cases later confirmed as true or false positive [40].

The incidence of neonatal B12D associated with maternal B12D has been estimated to be 32% in Western populations [41,42]. Three mothers from our cohort of children with severe, symptomatic B12D in infancy were diagnosed with autoimmune gastritis or pernicious anaemia, and one with a not further specified gastritis. The prevalence of autoimmune gastritis is estimated at 1–2% in the general population [43], but the condition is considered underdiagnosed [44]. Women are affected more frequently. Not only is autoimmune gastritis associated with other autoimmune diseases [43], but it impairs the functioning and distribution of immune cells. These dysfunctions can be corrected by treatment with Cbl [45]. Insufficient maternal B12 nutritional intake was the most frequent cause to which infant B12D was attributed by the clinicians. Insufficient B12 intake and declining B12 levels during pregnancy have been described in many populations [46]. In an Italian cohort with mixed ethnic background, B12 intake during pregnancy did not reach the here recommended 2.6 µmol/d in 59% of women [38].

Maternal B12D is not only associated with the risk for consecutive B12D in neonates or infants but with adverse pregnancy outcomes and maternal health risks. Low maternal B12 levels (<148 pmol/L) are associated with preterm birth, low birth weight [47], higher maternal blood glucose levels and increased risk for gestational diabetes [48]. B12D is an important cause of maternal anaemia during pregnancy and anaemia in pregnancy is associated with an increased risk of maternal morbidity, preterm birth and low birth weight [49].

Taking a medical history with a focus on diseases predisposing to or causing B12D and assessing B12 status as part of routine care during early pregnancy would open opportunities for interventions that might benefit both mother and child. Women with low B12 concentrations in early pregnancy should receive diagnostic workup for B12 resorption disorders or low nutritional supply. Prospective maternal screening for B12D during pregnancy would allow healthcare providers to inform about the importance of B12, to provide nutritional counselling and to prescribe B12 supplements during pregnancy and breastfeeding for mothers with B12D-relevant health issues or nutritional habits.

The B12 status of the mother, umbilical cord blood [50] and newborn B12-related parameters [16] correlate strongly with each other, but not every newborn of a B12-deficient mother will have B12D, and some newborns may have B12D despite adequate maternal B12 status [50]. These data suggest that the transplacental maternal-to-foetus transport rate of B12 is variable [51].

The efficacy of oral vitamin B12 supplementation during pregnancy has recently been reviewed. Optimisation of the maternal B12 status can reduce maternal and neonatal B12D, but the evidence for short- and long-term effects of B12 supplementation on mother and child is still uncertain and may vary significantly between populations [52].

Our data tentatively indicate that NBS for B12D may contribute to preventing an increase in clinically diagnosed severe B12D. However, the interpretation of this trend is limited by the incomplete reporting of cases, which precludes an accurate estimate of the true number of affected individuals. Future studies should therefore assess whether the observed pattern persists once data reflecting the full case spectrum become available. In addition, it deserves to be investigated whether the approach to supplement women at risk or with manifest B12D during pregnancy would, together with NBS, be more effective and economical in preventing severe, symptomatic B12D in infants and in reducing the rate of burdensome NBS recalls and workups.

This study has several limitations. The diagnosis of B12D was made by clinicians and did not follow a uniform algorithm, and the diagnostic criteria for B12D in children are generally not sufficiently standardised. The variability in diagnostic criteria as well as of the assays used in different institutions might undermine the comparability of cases, and potentially bias prevalence estimates and conclusions about NBS effectiveness. Furthermore, we must assume selection bias because it remains uncertain whether all cases of severe B12D have been ascertained by our retrospective questionnaire approach dating back over ten years, given the limited response rate from the centres. A reporting bias towards more memorable severe cases cannot be excluded. Material for second-tier testing was not available for all first-tier positives and second-tier tHcy testing was performed on DBS after variable times of storage.

Conclusions: B12 is related to multiple important methylation reactions. B12D can cause severe clinical symptoms, especially in infants, and should be prevented. NBS has a very limited detection rate of newborns who develop severe B12D during infancy; most of them have a normal NBS. However, it appears that NBS may contribute to a reduction in the incidence of cases through other mechanisms that have not yet been precisely quantified. The workup triggered by NBS recalls is costly and may place a strain on families during a particularly sensitive period. Maternal B12D is associated with pregnancy-related and general health risks for mother and child. The B12 status of newborns and their mothers are highly associated. Women should be screened for B12D routinely during pregnancy to be counselled and treated to promote a healthy pregnancy and breastfeeding period and to reduce the frequency of NBS recalls.

## Figures and Tables

**Table 1:** Sample characteristics.

Parameter	Mean/Median/Range	N
Birthweight z-score	−0.6/−0.6/−3.62–2.65	44
Length at birth z-score	−0.8/−0.8/−3.7–1.4	40
Head circumference at birth z-score	−0.2/−0.2/−2.8–1.7	38
Weight at diagnosis z-score	−1.3/−1.3/−4.5–2.3	46
Length at diagnosis z-score	−0.8/−0.7/−4.3–1.5	42
Head circumference at diagnosis z-score	−0.5/−0.6/−3.0–1.9	39
Further diagnoses (infant)		4
○ Central hypothyreosis		1
○ Multicystic kidney, pulmonary stenosis		1
○ Neonatal infection, basal ganglia calcification		1
○ Syndromatic anophthalmia and deafness		1
Infant diet at diagnosis		45
○ Fully breastfed		28
○ Breastfed and formula		3
○ Breastfed and complementary food		7
○ Breastfed and formula & complementary food		4
○ Formula only		2
○ Formula and complementary food		1
If complementary food		12
○ Includes food from animal origin		5
○ No food from animal origin		7
Maternal diet at diagnosis		34
○ mixed, including food from animal origin		23
○ vegetarian including egg and dairy products		5
○ vegan		6
History of maternal health problems		30
- None		9
- Maternal health problems		21
○ Autoimmune gastritis/pernicious anaemia		3
○ Gastritis (unspecified)		1
○ Anaemia (unspecified)		2
○ Vitamin B12 deficiency (unspecified)		2
○ Iron deficiency		1
○ Crohn’s disease		2
○ Ulcerative colitis		1
○ Hemicolectomy and small bowel resection		1
○ Multiple sclerosis		1
○ Hashimoto thyreoditis/hypothyroidism		3/2
○ Lactose intolerance and irritable bowel		1
○ Postpartal depression		1

**Table 2:** Biochemical parameters at diagnosis.

	B12 (ng/L)	HoloTC (pmol/L)	tHcy (µmol/L)	MMA in Blood (µg/L)	MMA in Urine (mmol/mol Creatinine)
N	42	14	25	21	36
Mean (SD)	193 (191)	59 (69)	48 (69)	494 (462)	250 (546)
Median (IQR)	121 (214)	32 (50)	12 (94)	387 (405)	50 (164)
Range	16–750	5–256	3–275	32–1758	0–2700

Significant correlations (two-tailed p) between the biochemical parameters at diagnosis and clinical symptoms are shown in Table 3.

## Data Availability

The raw data supporting the conclusions of this article will be made available by the authors on request.
